# Notes Relating to Botany, Collected from the Manuscripts of the Late Peter Collinson, Esq. F. R. S.

**Published:** 1811-12

**Authors:** Aylmer Bourke Lambert


### *Notes relating to Botany, collected from the Manuscripts of the late* Peter Collinson, Esq. F. R. S. *and communicated by* Aylmer Bourke Lambert, Esq. F. R. S. and A. S.; V. P. L. S.

(Trans. Linn. Soc. Vol. x.)

Being lately on a visit to John Cator, Esq. of Beckenham-place, and looking one day over his library, amongst a collection of books left him by his uncle, who married the daughter of the celebrated Peter Collinson, I discovered several which had formerly belonged to that eminent naturalist. One of them was his own copy of Miller's Gardener's and Botanist's Dictionary, the last edition, published by the author, with the following note at the bottom of the title-page: "The gift of my old friend the author to P. Collinson, F. R. S." This book contains a great deal of his manuscript notes relating to the plants cultivated in those days, both in his own gardens and in those of the most celebrated of his contemporaries; with a complete catalogue of the plants he had cultivated in his garden at Mill-hill, and a list of all those which he had himself introduced into this country from Russia, Siberia, America, and other parts of the world; also some original letters from Dillenius, Miller, Bartram, and others; and a short account of his own life, which appears not to have been known to his biographers. Mr. Cator having obligingly permitted me to take a copy of the whole, I now submit to the Linnean Society those parts which I think most worthy of their notice. A. B. L.

I was born in the house against Church-alley, Clement's Lane, Lombard-street, from whence my parents removed into Grace-church-street, where I have now lived many years. [July 18th, 1764.] Gardening and gardeners have wonderfully increased in my memory. Being sent at two years old to be brought up with my relations at Peckham in Surry, from them I received the first liking to gardens and plants. Their garden was remarkable for fine cut greens, the fashion of those times, and for curious flowers. I often went with them to visit the few nursery gardens round London, to buy fruits, flowers, and clipt yews in the shapes of birds, dogs, men, men, ships, &c. For these Mr. Parkinson in Lambeth was very much noted; and he had besides a few myrtles, oleanders, and other evergreens. This was about the year 1712. At that time Mr. Wrench, behind the earl of Peterborough's at Parson's Green, near Chelsea, famous for tulip-trees, began the collecting of evergreens, arbutuses, phillyreas, &c.; and from him came the gold and silver hedgehog-holly, being accidental varieties from the hedgehog variety of the common holly. He gave rewards to encourage people to look out for accidental varieties from the common holly: and the saw-leaved holly was observed by these means, and a variegated holly goes by his name to this day. He and Parkinson died about the year 1724. Contemporary with them were Mr. Derby and Mr. Fairchild; they had their gardens on each side the narrow alley leading to Mr. George Whitmore's, at the further end of Hoxton. As their gardens were small, they were the only people for exotics, and had many stoves and green-houses for all sorts of aloes and succulent plants; with oranges, lemons, and other rare plants. At the other end of the town were two famous nurserymen, Furber and Gray, having large tracts of ground in that way, and vast stocks: for the taste of gardening increased annually. Doctor Compton, bishop of London, was a great lover of rare plants; as well such as came from the West Indies as from North America, and had the greatest collection then in England. After his death the see was filled by bishop Robinson, a man destitute of any such taste; who allowed his gardener to sell what he pleased, and often spoiled what he could not otherwise dispose of. Many fine trees, come to great maturity, were cut down to make room for produce for the table.

The above mentioned gardeners, Furber and Gray, availed themselves of making purchases from this noble collection, and augmented their nurseries with many fine plants not otherwise to be procured.

Brompton Park was another surprising nursery of all the varieties of evergreens, fruits, &c. with a number of others all round the town; for, as the taste increased, nursery gardens flourished.

Mr. Hunt, at Putney, and Mr. Gray, are now living, aged about 70. But more modern cultivators are the celebrated James Gordon at Mile-end, whom for many years, from my extensive correspondence, I have assisted with plants and seeds, and who, with a sagacity peculiar to himself, has raised a vast variety of plants from all parts of the world; and the ingenious Mr. Lee of Hammersmith, who, had he the like assistance, would be little behind him. Mr. Miller of the Physic Garden, Chelsea, has made his great abilities well known by his works, as well as his skill in every part of gardening, and his success in raising seeds procured by a large correspondence. He has raised the reputation of the Chelsea garden so much, that it excels all the gardens in Europe for its amazing variety of plants of all orders and classes, and from all climates, as I beheld with much delight this 19th of July, 1764.

October 3d, 1759, after nine years absence from Goodwood after the death of my intimate friend the late duke of Richmond, I accompanied the present duchess there, and to my agreeable surprise found the hardy exotic trees much grown. There were two fine great magnolias about twenty feet high in the American grove that flowered annually. (My tree flowered this year, 1760, that I raised from seed about twenty years before.) Some of the larches measured near the ground seventeen inches round, the rest fourteen inches and a half. I saw a larch of the old duke's planting cut down, that in twenty-five years was above fifty feet high, and cut into planks above a foot in diameter, and above twenty feet long: but there were some larches of the same date seventy feet high. They grow wonderfully in chalky soil.

October 30th, 1762, the young lord Petre came of age. The late lord Petre, his father, died July 2d, 1742: he was my intimate friend, the ornament and delight of the age he lived in. He went from his house at Ingatestone in Essex, to his seat at Thorndon-hall in the same county, to extend a large row of elms at the end of the park behind the house. He removed in the spring of the year 1734, being the 22d of his age, twenty-four full-grown elms about sixty feet high and two feet diameter. All grew finely, and now are not known from the old trees they were planted to match. In the year 1738 he planted the great avenue of elms up the park from the house to the esplanade. The trees were large, perhaps fifteen or twenty years old: On each side the esplanade, at the head or top of the park, he raised two mounts, and planted all with evergreens in April and May 1740. In the center of each mount was a large cedar of Lebanon of twenty years growth, supported by four larches of eleven years growth. On the same area on the mount were planted four smaller cedars of Lebanon aged twenty years each, supported by four larches aged six years. On the sides Virginian red cedars of three years growth, mixed with other evergreens, which now (anno 1760) make an amazingly fine appearance.

In the years 1741 and 1742, from this very nursery, he planted out forty thousand trees of all kinds, to embellish the woods at the head of the park on each side of the avenue to the lodge, and round the esplanade. It would occupy a large large work to give a particular account of his building and planting. His stoves exceed in dimensions all others in Europe. He dying, his vast collection of rare exotic plants, and his extensive nursery, were soon dispersed.

I paid to John Clarke for a thousand cedars of Lebanon, June the 8th, 1761, seventy-nine pounds six shillings, in behalf of the duke of Richmond. These thousand cedars were planted at five years old, in my sixty-seventh year, in March and April, anno 1761.

In September, 1761, I was at Goodwood, and saw these cedars in a thriving state.

This day, October 20th, 1762, I paid Mr. Clarke for another large parcel of cedars for the Duke of Richmond. It is very remarkable that Mr. Clarke, a butcher at Barnes, conceived an opinion that he could raise cedars of Lebanon from cones from the great tree at Hendon-place. He succeeded perfectly; and annually raised them in such quantities, that he supplied the nurserymen, as well as abundance of noblemen and gentlemen, with cedars of Lebanon: and he succeeded not only in cedars, but he had a great knack in raising the small magnolia, Warner's Cape jessamine, and other exotic seeds. He built a large stove for pine apples, &c.

Any person who has curiosity enough may go to Goodwood in Sussex, and see the date and progress of those cedars, which were at planting five years old. The duke's father was a great planter; but the young duke much exceeds him, for he intends to clothe all the lofty naked hills above him with evergreen woods. Great portions are already planted, and he annually raises infinite numbers in his nurseries from seeds of pines, firs, cedars, and larches.

In the duke of Argyle's wood stands the largest New-England or Weymouth pine. This, and his largest cedars of Lebanon now standing, were all raised by him from seed in the year 1725 at his seat at Whitton near Hounslow.

This spring, 1762, all the duke of Argyle's rare trees and shrubs were removed to the princess of Wales's garden at Kew, which now excels all others, under the direction of lord Bute.

Mr. Vernon, Turkey merchant at Aleppo, transplanted the weeping-willow from the river Euphrates, brought it with him to England, and planted it at his seat at Twickenham-park, where I saw it growing anno 1748. This is the original of all the weeping-willows in our gardens.*

---

* This is the first authentic account we have had of its introduction; the story of its being raised from a live twig of a fruit-basket, received from Spain by Pope, being only on newspaper autho-

 rity

October the 18th, 1765, I went to see Mr. Rogers's vineyard, all of Burgundy grapes, and seemingly all perfectly ripe. I did not see a green half-ripe grape in all this great quantity. He does not expect to make less than fourteen hogsheads of wine. The bunches and fruit are remarkably large, and the vines very strong. He was formerly famous for ranunculuses.

October 18th, 1765, I visited Mrs. Gaskry, at Parson's Green, near Fulham. This long, hot, dry summer has had a remarkably good effect on all wall fruits. Apricots, peaches, and nectarines, ripened much earlier than usual, and have been excellent: but the most remarkable was the plenty of pomegranates, near two dozen on each tree, of a remarkable size and fine ruddy complexion, of the size of middling oranges. One that was split shewed the redness and ripeness within.

John Buxton, esq. of Shadwell, near Thetford, in Norfolk, from the acorns of 1762, sowed or planted on forty-two acres of land 120 bushes, containing as near as can be computed 1,432,320 acorns; which is nearly 34,103 acorns on each acre. For this Mr. Buxton had a present of a gold medal from the Society of Arts, &c. Years or ages hence it may be worth a journey to go and observe the progress of vegetation in the dimensions and heights of this famous plantation, whose beginning is so certainly known.

By a letter (November 28th, 1762), from Thomas Knowlton, gardener to the duke of Devonshire, at his seat of Londesburgh near York, and director to his grace's new kitchen-garden, stoves, &c. at Chatsworth, I am informed that the duke of Devonshire is now sowing seventy quarters of acorns, that is, 560 bushels: an immense quantity: but this year there was the greatest crop of acorns ever remembered. Besides this vast sowing, some hundred thousands of young seedling oaks are planting out this winter: between forty and fifty men are employed about this work. In the year 1761, as many oaks were transplanted from the nursery, of two, three, and four years old.

---

rity so late as August, 1801.—See Miller's Dictionary by Martyn. —A. B. L.

Sir Thomas Vernon of London, Knight, and some time member for that city, died in 1705, leaving two sons. Henry, the eldest, died unmarried at Aleppo in Syria, aged 31; his monument is in St. Stephen's church, Coleman-street. Thomas Vernon, the second son, resided at Twickenham-park, Middlesex.

The above communicated to me by Sir William A'Court, bart. nephew to Mr. Vernon.—A. B. L.

1761.

1761. Our last winter, if it may be called so, exceeded for mildness 1759. The autumnal flowers were not gone before spring began in December with aconites, snowdrops, polyanthuses, &c. and continued without any alloy of intervening sharp frosts, all January, except two or three frosty nights and mornings: a more delightful season could not be enjoyed in southern latitudes. In January and February my garden was covered with flowers.

This summer, 1762, I was visiting Mr. Wood, of Littleton, Middlesex. He showed me a curiosity which surprized me. On a little slender twig of a peach-tree about four inches long, that projected from the wall, grew a peach, and close to it, on the other side of the twig, a nectarine. This Mr. Miller also assured me he had himself known, although not mentioned here (in his Dictionary); and another friend* assured me that he had a tree which produced the like in his garden at Salisbury: but this I saw myself, and it induces me to think that the peach is the mother of the nectarines; the latter being a modern fruit, as there is no Greek or Latin name for it.

Copied from my nephew Thomas Collinson's Journal of his Travels, 1754.—" In the reign of Queen Elizabeth, anno the first orange and lemon-trees were introduced into England by two curious gentlemen, one of them Sir Nicholas Carew, at Bedington, near Croydon, in Surrey." (The title is lately extinct, anno 1763.) These orange-trees were planted in the natural ground; but against every winter an artificial covering was raised for their protection. I have seen them some years ago in great perfection. But this apparatus going to decay, without due consideration a green-house of brick-work was built all round them, and left on the top uncovered in the summer. I visited them a year or two after, in their new habitation, and to my great concern found some dying, and all declining; for, although there were windows on the south-side, they did not thrive in their confinement; but being kept damp with the rains, and wanting a free, airy, full sun all the growing months of summer, they languished, and at last all died.

A better fate has hitherto attended the other fine parcel of orange-trees, &c. brought over at the same time by Sir Ro-

---

* I well knew the gentleman here alluded to, Dr. Hancock of Salisbury, who assured me of this fact; and a drawing showing both the fruits on the same branch is now in the possession of H. P. Wyndham, esq. of Salisbury.

Dr. Hancock told me that he had the tree taken up to send to the earl of Harburgh, but it was killed by removing.—A. B. L.

bert Mansell, at Margam; late Lord Mansell's, now Mr. Talbot's, called Kingsay-castle, in the road from Cowbridge to Swansey, in South Wales. My nephew counted eighty trees of citrons, limes, burgamots, Seville and China orange-trees, planted in great cases all ranged in a row before the green-house. This is the finest sight of its kind in England. He had the curiosity to measure some of them. A China orange measured in the extent of its branches fourteen feet. A Seville orange was fourteen feet high, the case included, and the stem twenty-one inches round. A China orange twenty-two inches and a half in girth.

July 11th, 1777. I visited the orangery at Margam in the year 1766, in company with Mr. Lewis Thomas, of Eglews Nynngt in that neighbourhood, a very sensible and attentive man, who told me that the orange trees, &c. in that garden were intended as a present from the king of Spain to the king of Denmark; and that the vessel in which they were shipped being taken in the Channel, the trees were made a present of to Sir R. Mansell.

December 10th, 1765. A few days ago died my friend Mr. Bennet, who was very curious and industrious in procuring seeds and plants from abroad. He had a garden behind the Shadwell water-works near the spot where he lived, and built several very handsome stoves at a great expence, filling them with fine exotics of all kinds; but the erecting a fire-engine to raise the water so hurt his plants by the smoke, that he removed to a large garden of two or three acres, in the fields at the back of Whitechapel laystalls. Here he built a large house for pines and other rare exotics, which he left well stocked. In this garden he raised water melons to a great size and perfection; I have told above forty lying ripe on the ground. They were raised in frames, and transplanted out under bell-glasses. A basket of these melons was sent to the king. Mr. Bennet had besides a great collection of hardy-ground plants. His garden and all his plants were sold by auction, April 14, 1766.

The seeds of the rhubarb with broad curled leaves were first raised by me. They were sent by Dr. Amman, professor of botany at Petersburg, whose father-in-law was Russian governor of the province near which the rhubarb grows. The seed of that with long narrow curled leaves was sent by the Jesuits in China to my friend Dr. Tanches, at Petersburg, by the Russian caravan, and he sent it to me.

Lord Rochefort, our ambassador in Spain, in a letter dated Madrid, November 1765, says, that in the parts where he had been, there are very few forest-trees worth notice; but the ilexes about the Escurial are fine. One sort produces acorns of of a monstrous size, which they eat in Spain at their best tables, and they are as sweet as chesnuts.

May 17th, 1761. I was invited by Mr. Sharp, at South Lodge, on Enfield Chase, to dine, and see the Virginia dog-wood (*Cornus florida*). The calyx of the flowers is as large as those figured by Catesby, and (what is remarkable) this is the only tree that bears these flowers amongst many hundreds that I have seen: it began to bear them in May, 1759.

Anno 1747. Raised a new species of what appears to be a three-thorned acacia, from seeds from Persia, that came with Azad or Persian hornbeam, given me by Mr. Baker: it thrives well in my garden. I gave seed to Mr. Gordon, and he also raised it.

The eastern hornbeam (Miller's Dictionary, edition 8th), was raised from seed given to me, which came from Persia by the name of *Azad*. I gave it to Mr. Gordon, gardener at Mile-End, who was so fortunate at to have it come up anno 1747, and from him my garden and other gardens have been supplied. There is a large tree in my field at Hendon, Middlesex.

Mr. Miller is greatly mistaken in saying the Arundo No. 2, or *Donax*, dies down every year. In my garden the stalks have continued for some years making annually young green shoots from every joint, and bear a handsome tassel of flowers. The first time I ever saw it in flower was September 15th, 1762. This very long hot dry season has made many exotics flower.

*Donax seu Arundo* flowered this year also (1762) at Mr. Gordon's at Mile-End.

October the 22d, 1746, I received the first double Spanish broom that was in England, sent me by my friend Mr. Brewer at Nuremberg: it cost there a golden ducat; and, being planted in a pot nicely wickered all over, came from thence down the river Elbe to Hamburg, from whence it was brought by the first ship to London. I inarched it on the single-flowered broom, and gave it to Gray and Gordon, gardeners, and from them all have been supplied.

Anno 1756. Some roots of Siberian martagon sent me by Mr. Demidoff, proprietor of the Siberian iron mines, flowered for the first time, May 24, 1756. The flower is but little reflexed, and is, I think, the nearest to black of any flower that I know.

In the year 1727, my intimate friend Sir Charles Wager, first lord of the admiralty, brought plants from Gibraltar-Hill, of the *Linaria procumbens Hispanica flore flavescente pulchrè striato, labiis nigro-purpureis*, which I have yet in my garden, anno 1761; and at the same time he brought the the broad-leaved *Teucrium*, and a species of periwinkle, neither of which were in our gardens before; and some roots of what is called *Hyacinths of Peru.*

In the year 1756, the famous tulip-tree in Lord Peterborough's garden at Parson's Green, near Fulham, died. It was about seventy feet high, the tallest tree in the ground, and perhaps a hundred years old, being the first tree of the kind that was raised in England. It had for many years the visitation of the curious to see its flowers, and admire its beauty, for it was as straight as an arrow, and died of age by a gentle decay. But it was remarkable, that the same year that this died, a tulip-tree which I had given to Sir Charles Wager flowered for the first time in his garden, which was opposite Lord Peterborough's. This tulip-tree I raised from seed, and it was thirty years old when it flowered.

April 8th, 1749. I removed from my house at Peckham, Surrey, and was for two years in transplanting my garden to my house at Mill-Hill, called Ridgeway-House, in the parish of Hendon, Middlesex.

Anno 1751. I raised the China or paper mulberry from seed given me by Dr. Mortimer.

